# Supplementary material for: Characterization of the C4 proteins encoded by okra-infecting geminiviruses in India
Source: BMC Plant Biol. 2026 Jul 7;26:1658. doi: 10.1186/s12870-026-09348-2 (PMC13621717; doi:10.1186/s12870-026-09348-2)

Fig. 5a

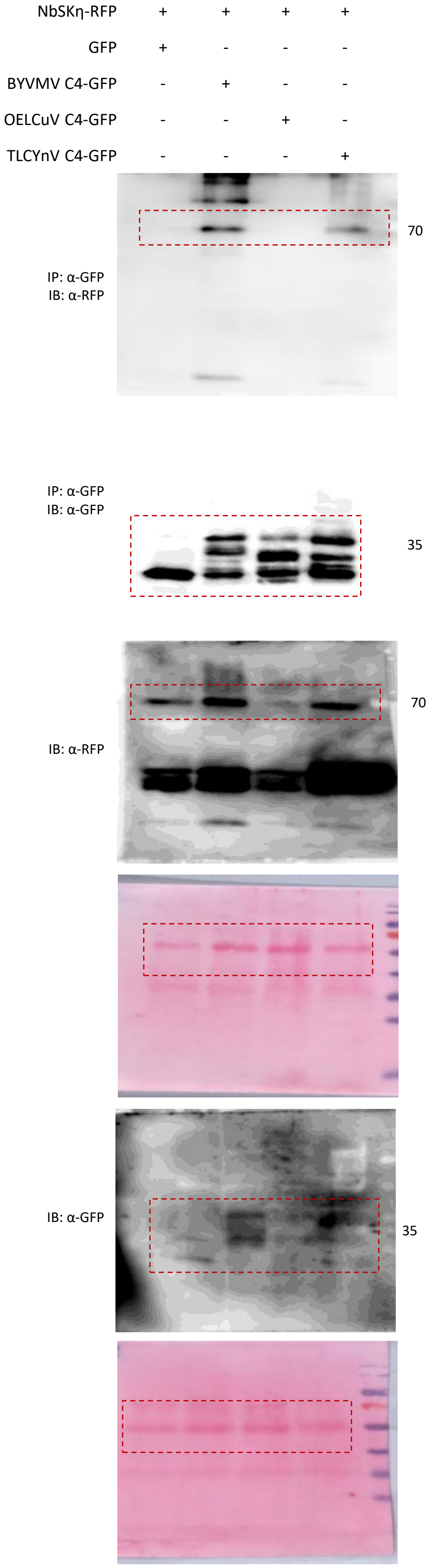

Fig. S3a

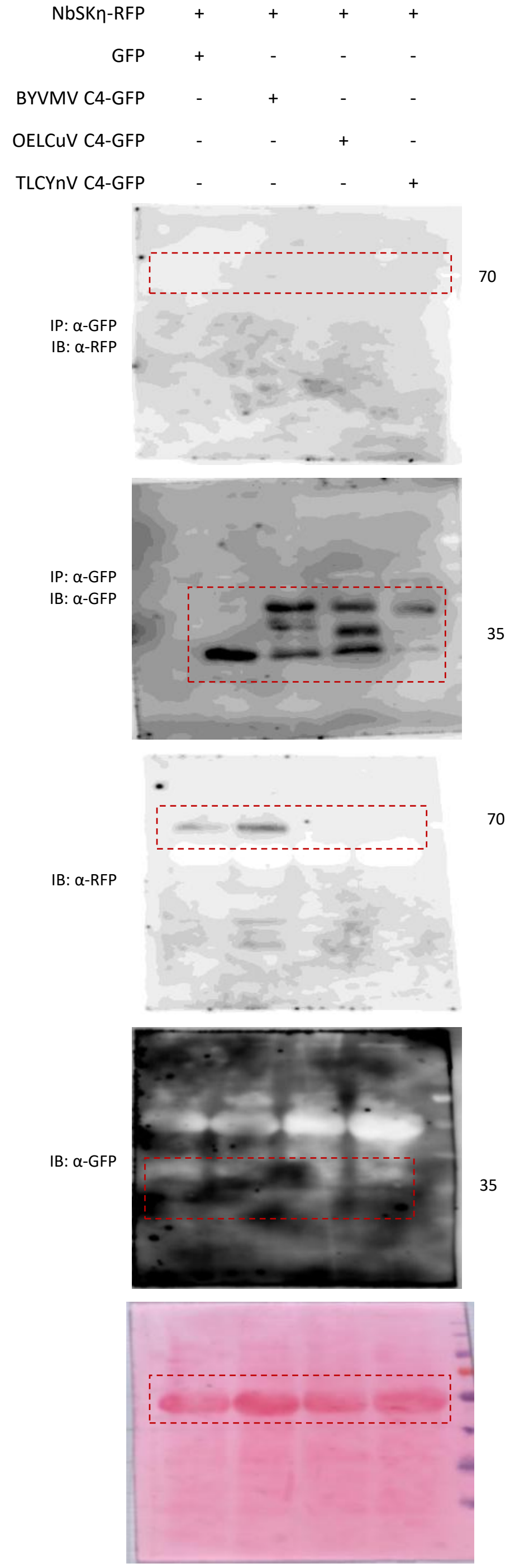

Fig. S3a

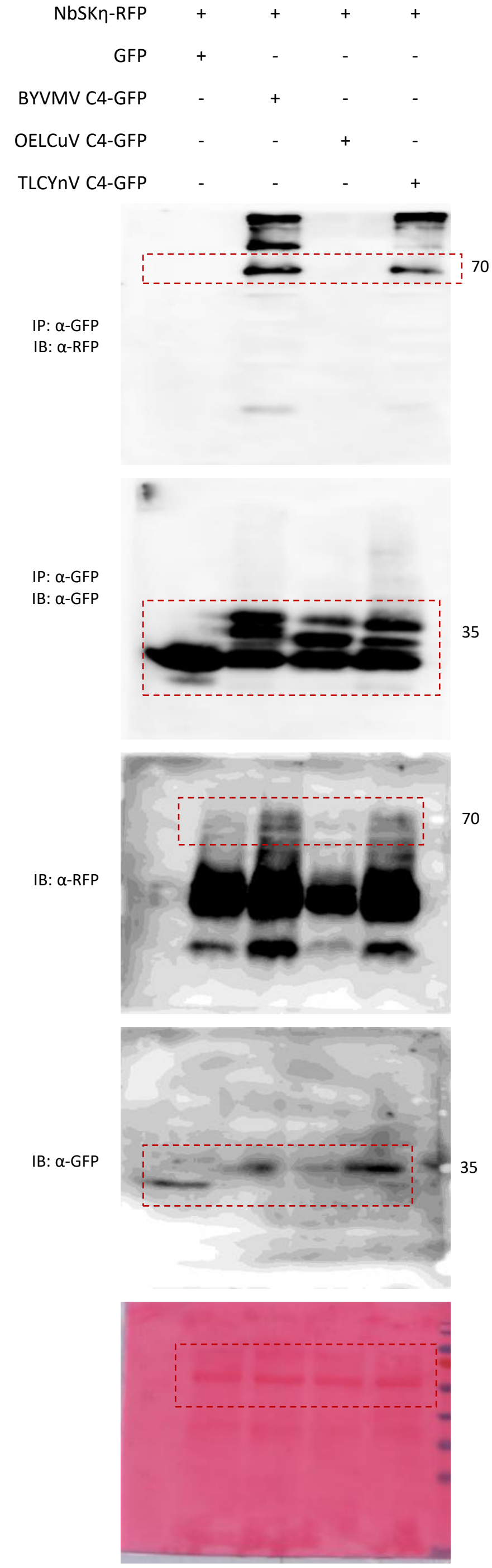

Fig. 5d

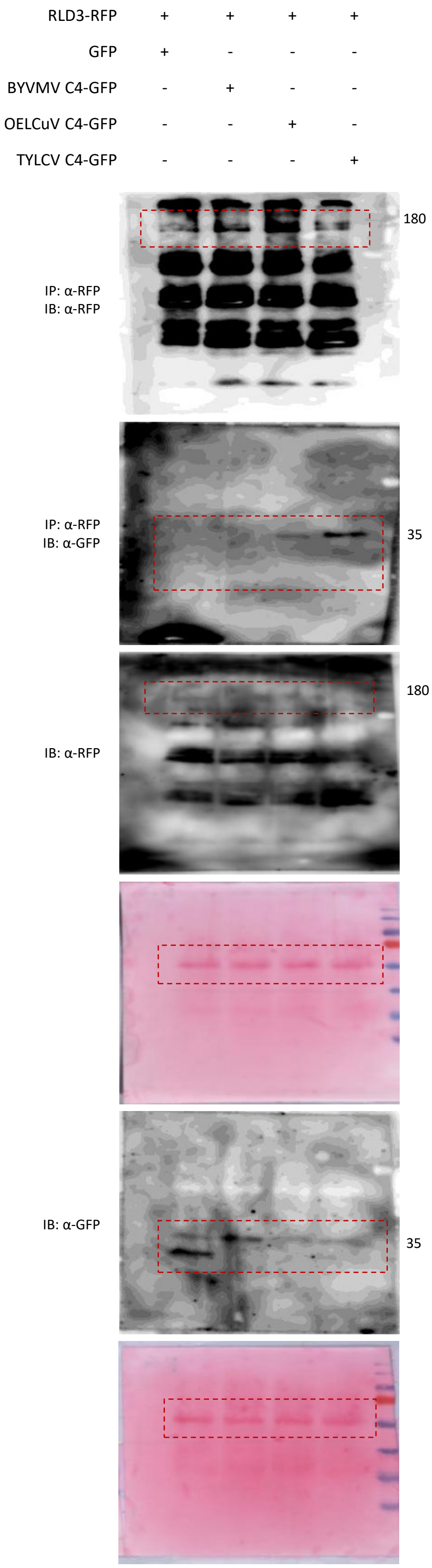

Fig. S3b

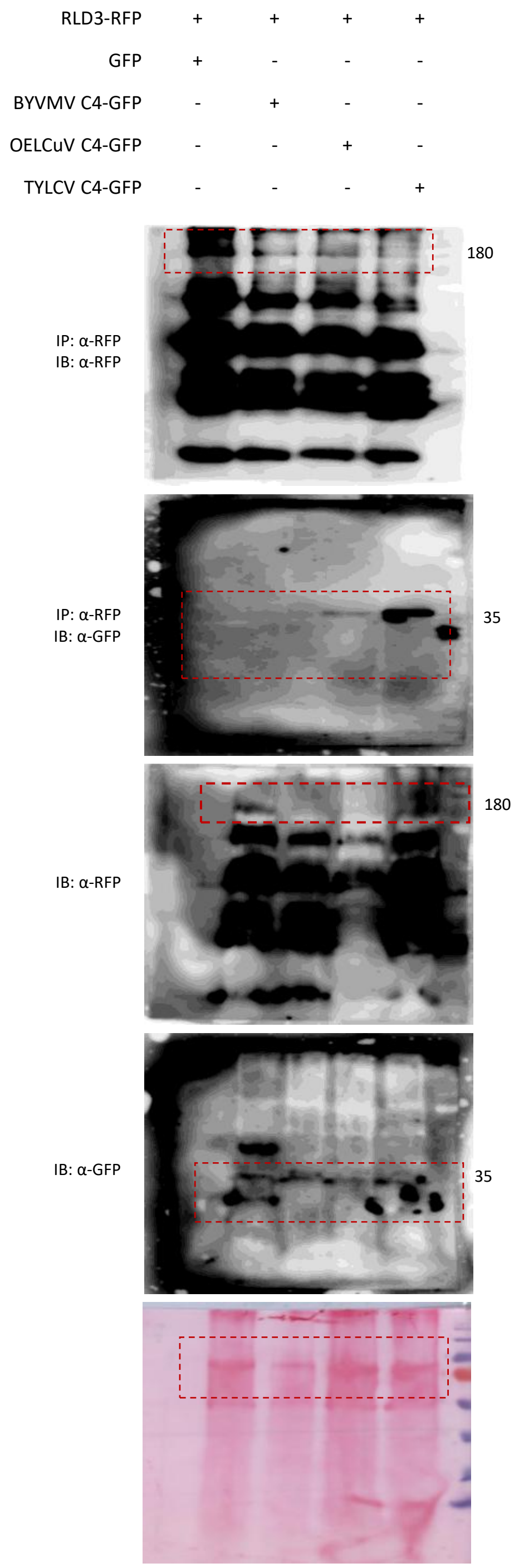

Fig. S3b

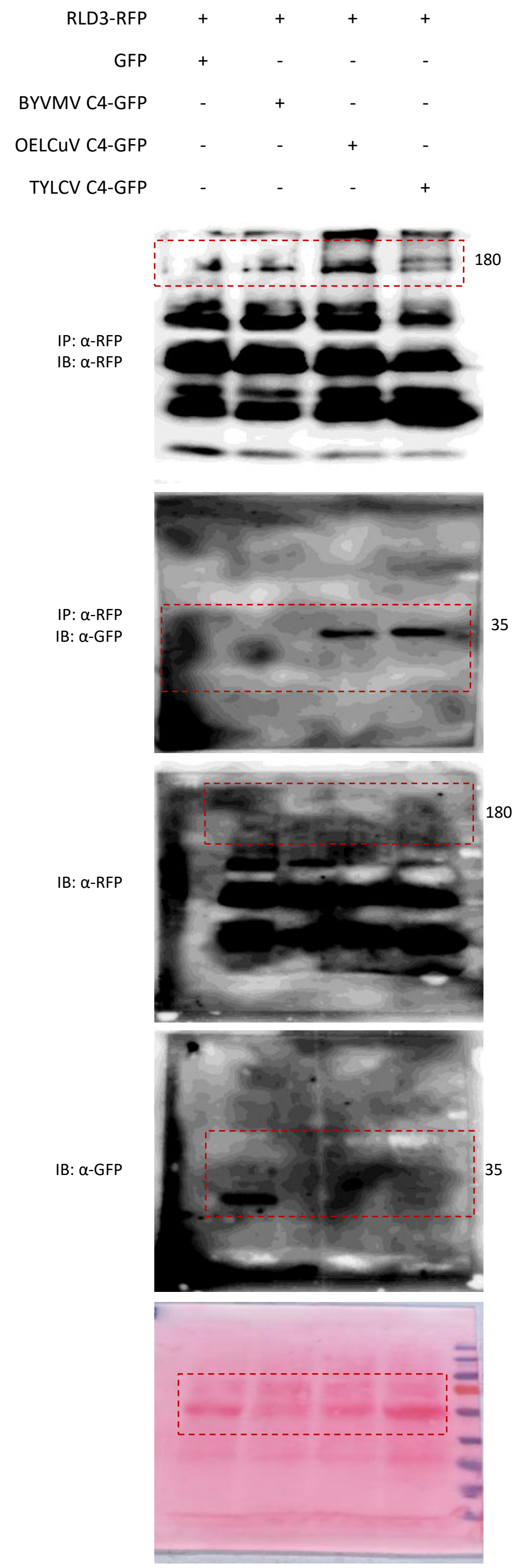

Fig. 7a

BAM1-RFP	+	+	+	+
GFP	+	-	-	-
BYVMV C4-GFP	-	+	-	-
OELCuV C4-GFP	-	-	+	-
TYLCV C4-GFP	-	-	-	+

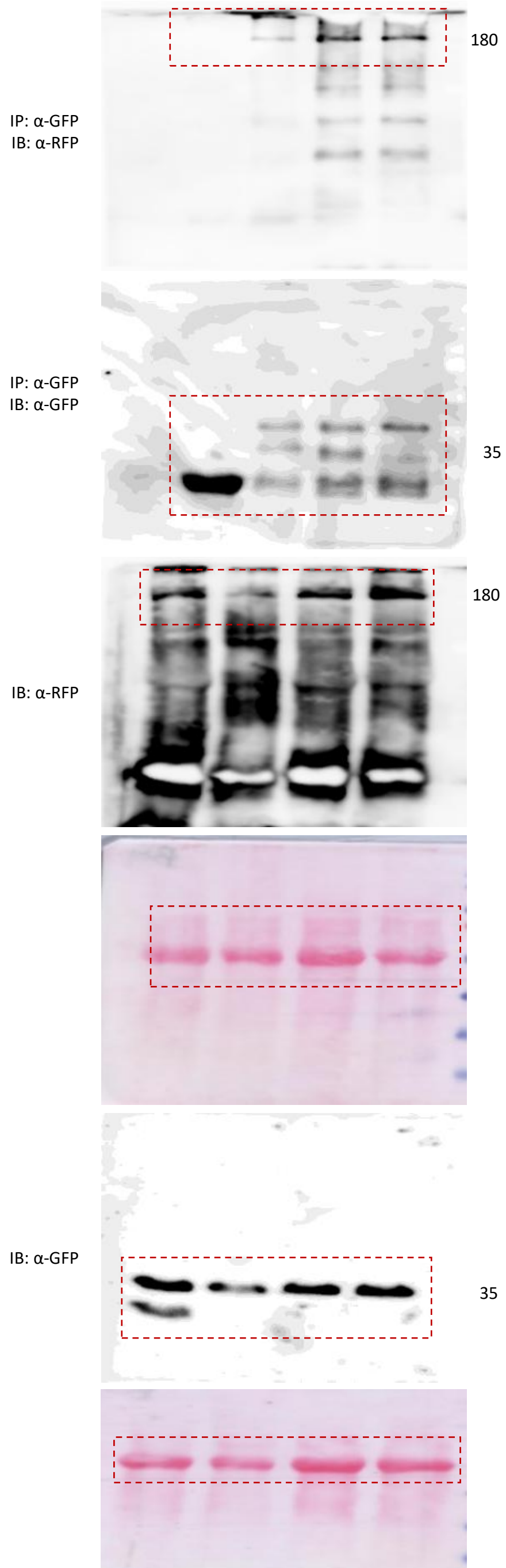

Fig. S3c

BAM1-RFP	+	+	+	+
GFP	+	-	-	-
BYVMV C4-GFP	-	+	-	-
OELCuV C4-GFP	-	-	+	-
TYLCV C4-GFP	-	-	-	+

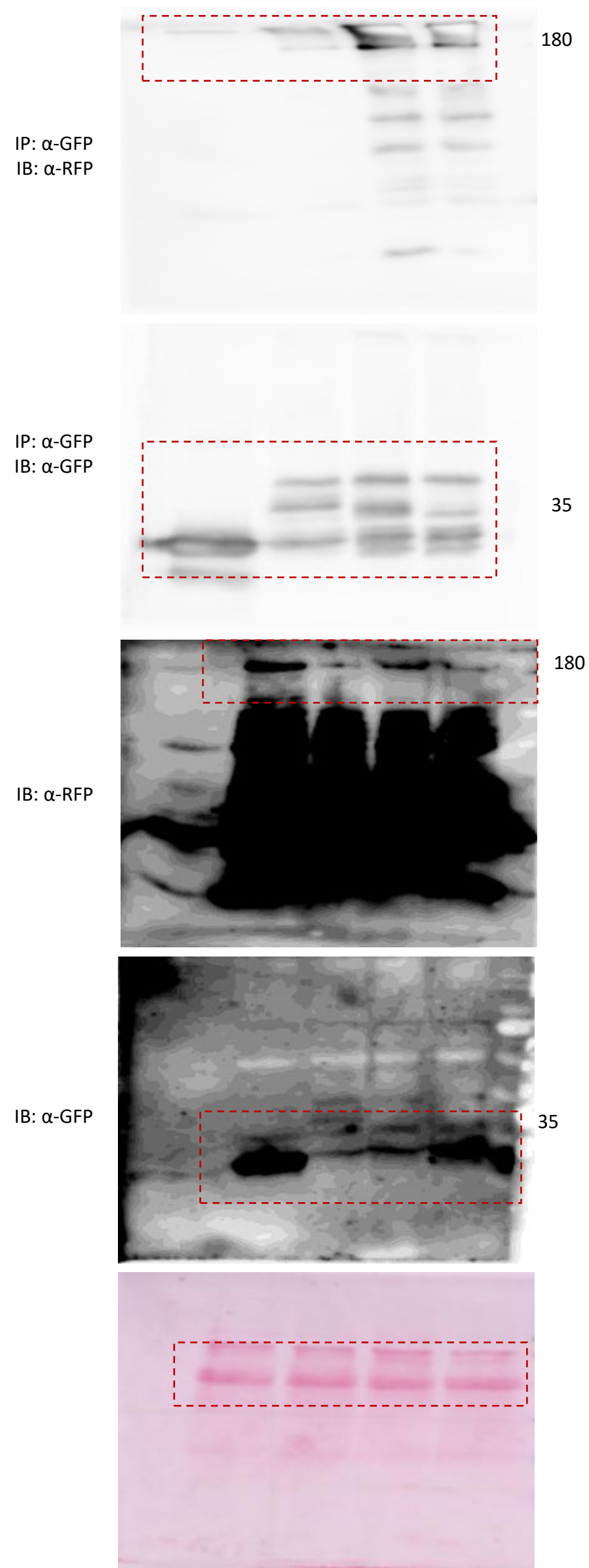

Fig. S3c

BAM1-RFP	+	+	+	+
GFP	+	-	-	-
BYVMV C4-GFP	-	+	-	-
OELCuV C4-GFP	-	-	+	-
TYLCV C4-GFP	-	-	-	+

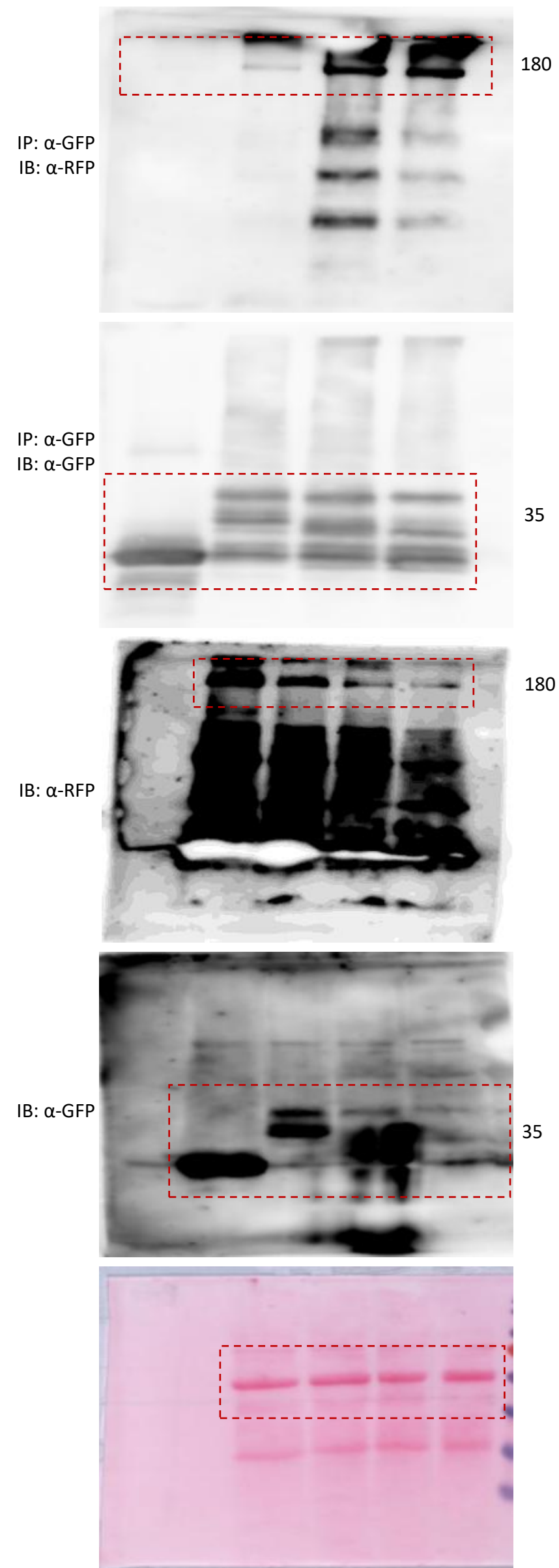

Supplement: Supplementary file 1 [file 12870_2026_9348_MOESM2_ESM.pdf]
