# Supplementary material for: Characterization of the C4 proteins encoded by okra-infecting geminiviruses in India
Source: BMC Plant Biol. 2026 Jul 7;26:1658. doi: 10.1186/s12870-026-09348-2 (PMC13621717; doi:10.1186/s12870-026-09348-2)
Supplement: Supplementary file 2 [file 12870_2026_9348_MOESM3_ESM.pdf]

SUPPLEMENTARY MATERIALS

Characterization of the C4 proteins encoded by okra-infecting geminiviruses in India

Aparna Chodon[1,2], Laura Medina-Puche[2], Hua Wei[2], Gopal Pandi[1]*, Rosa Lozano-Durán[2,3]*

[1]Department of Plant Biotechnology, School of Biotechnology, Madurai Kamaraj University, Madurai, Tamil Nadu 625021, India

[2]Department of Plant Biochemistry, Centre for Plant Molecular Biology (ZMBP), Eberhard Karls University, Tübingen 72076, Germany.

[3]Cluster of Excellence GreenRobust, Eberhard Karls University, Tübingen 72076, Germany.

*Co-corresponding authors: pgopal.biotech@mkuniversity.ac.in; rosa.lozano-duran@zmbp.uni-tuebingen.de

SUPPLEMENTARY TABLES

Supplementary table 1. Primers used in this work.

SUPPLEMENTARY FIGURES

SUPPLEMENTARY FIGURE 1

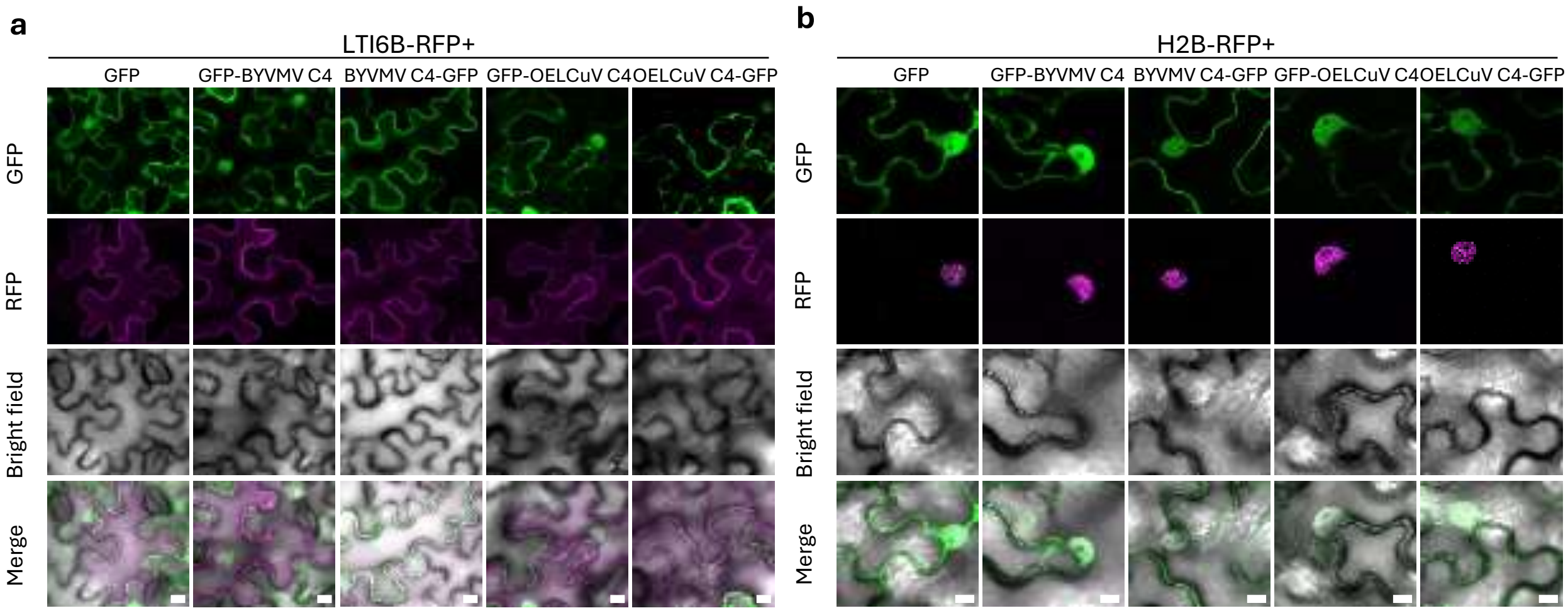

Supplementary figure 1. Colocalization analysis of GFP-fused C4 proteins in *N. benthamiana* epidermal cells. (a) Maximum projection of z-stack images of GFP-fused C4 proteins and the plasma membrane marker LTI6B-RFP in *N. benthamiana* leaves co-infiltrated with *A. tumefaciens* carrying constructs to express LTI6B-RFP and GFP fused proteins, or free GFP as a negative control. Images correspond to the single-plane images in Figure 3A. (b) Colocalization analysis of GFP-fused C4 proteins and the nuclear marker H2B-RFP. *A. tumefaciens* carrying constructs to express free GFP, or GFP fused C4 proteins were infiltrated in transgenic *N. benthamiana* plants stably expressing H2B-RFP. Images were taken at 2 days post-inoculation (dpi). Scale bar: 10 µm.

SUPPLEMENTARY FIGURE 2

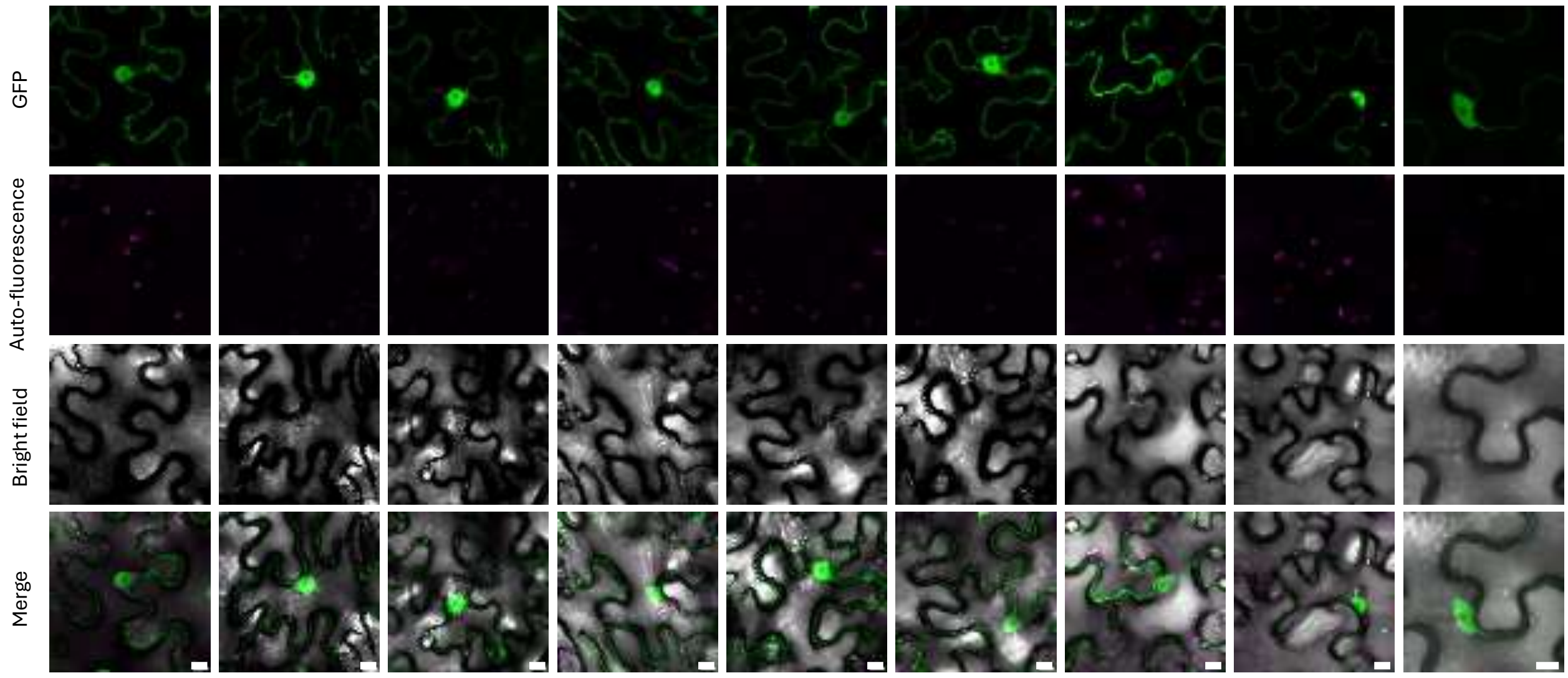

Supplementary figure 2. Subcellular localization of free GFP in *N. benthamiana* leaves. *N. benthamiana* leaves infiltrated with *A. tumefaciens* carrying a construct to express free GFP. Images were taken at 2 days post-inoculation (dpi). Scale bar: 10 µm.

SUPPLEMENTARY FIGURE 3

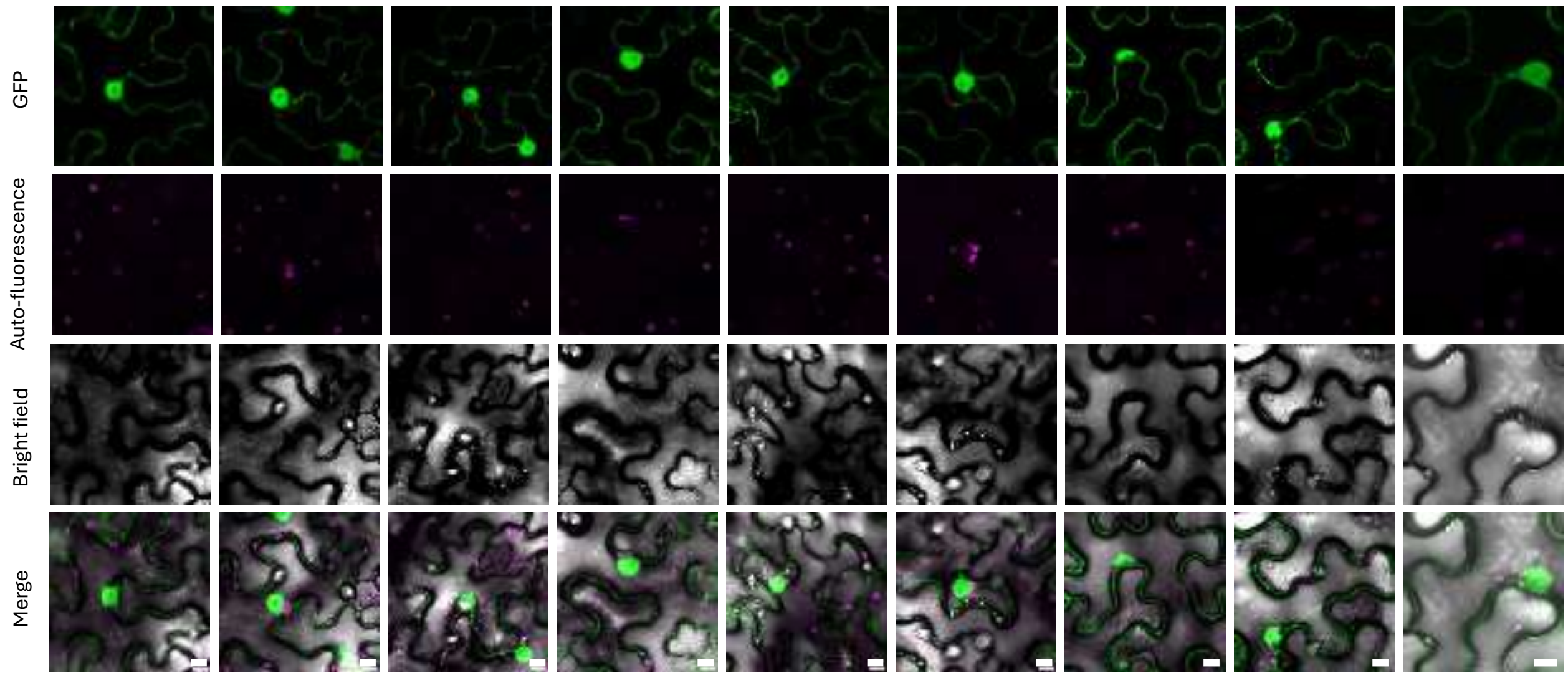

Supplementary figure 3. Subcellular localization of GFP-BYVMV C4 in *N. benthamiana* leaves. *N. benthamiana* leaves infiltrated with *A. tumefaciens* carrying a construct to express GFP-BYVMV C4. Images were taken at 2 days post-inoculation (dpi). Scale bar: 10 μm.

SUPPLEMENTARY FIGURE 4

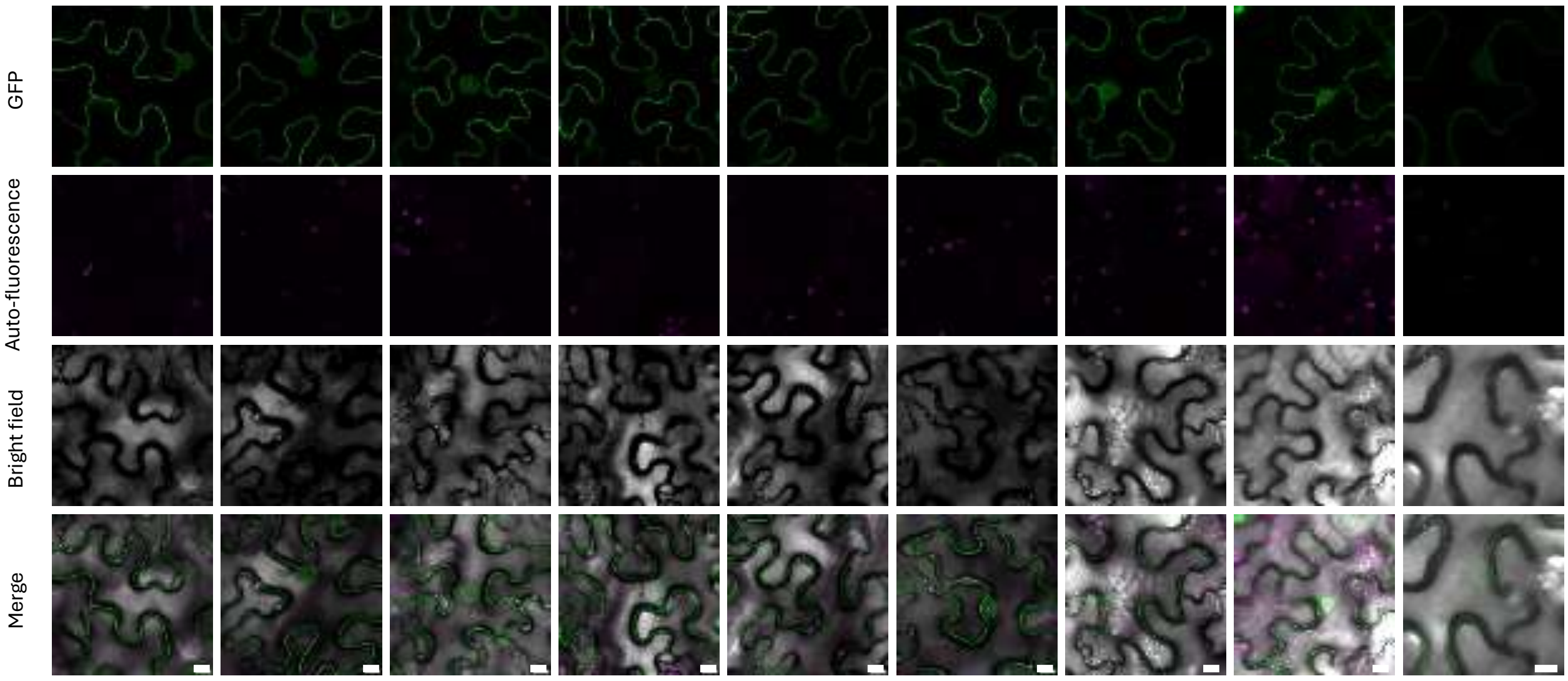

Supplementary figure 4. Subcellular localization of BYVMV C4-GFP in *N. benthamiana* leaves. *N. benthamiana* leaves infiltrated with *A. tumefaciens* carrying a construct to express BYVMV C4-GFP. Images were taken at 2 days post-inoculation (dpi). Scale bar: 10 μm.

SUPPLEMENTARY FIGURE 5

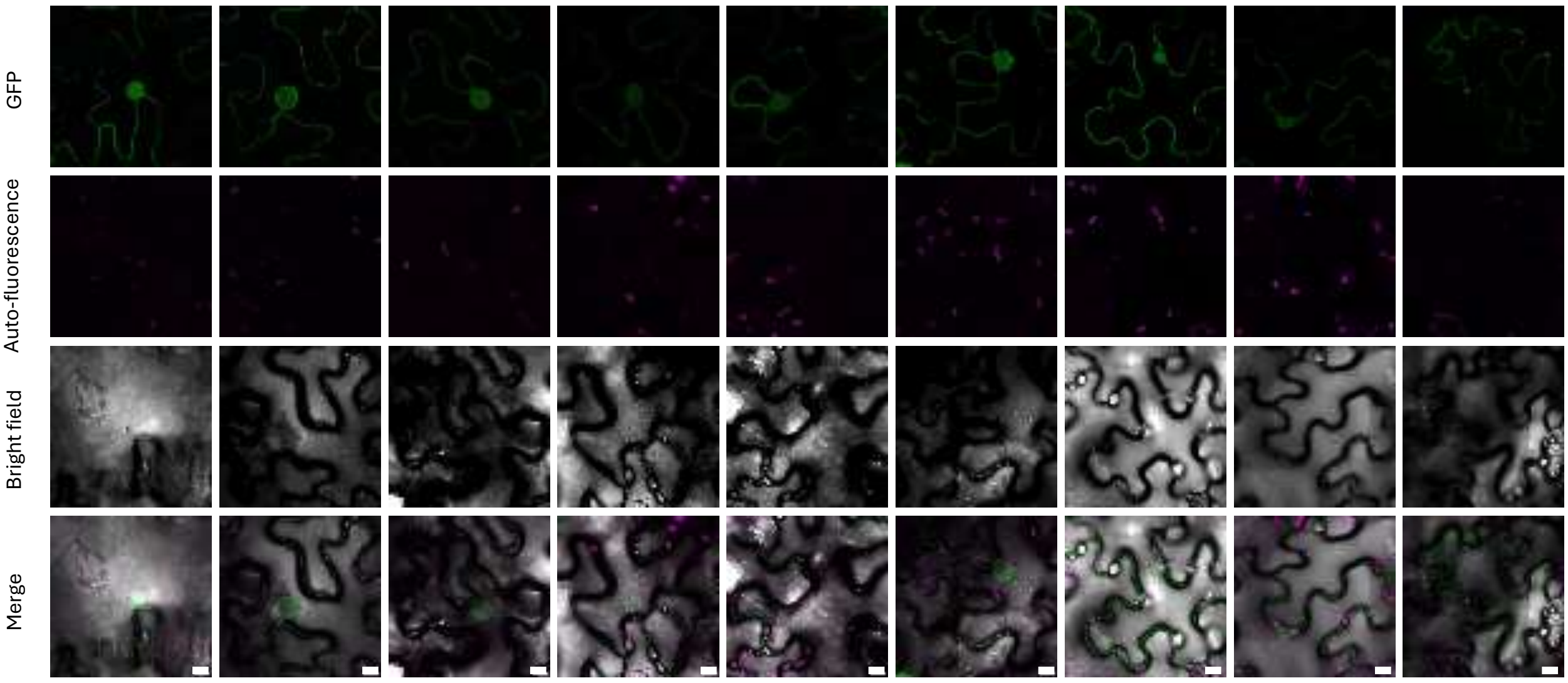

Supplementary figure 5. Subcellular localization of GFP-OELCuV C4 in *N. benthamiana* leaves. *N. benthamiana* leaves infiltrated with *A. tumefaciens* carrying a construct to express GFP-OELCuV C4. Images were taken at 2 days post-inoculation (dpi). Scale bar: 10 μm.

SUPPLEMENTARY FIGURE 6

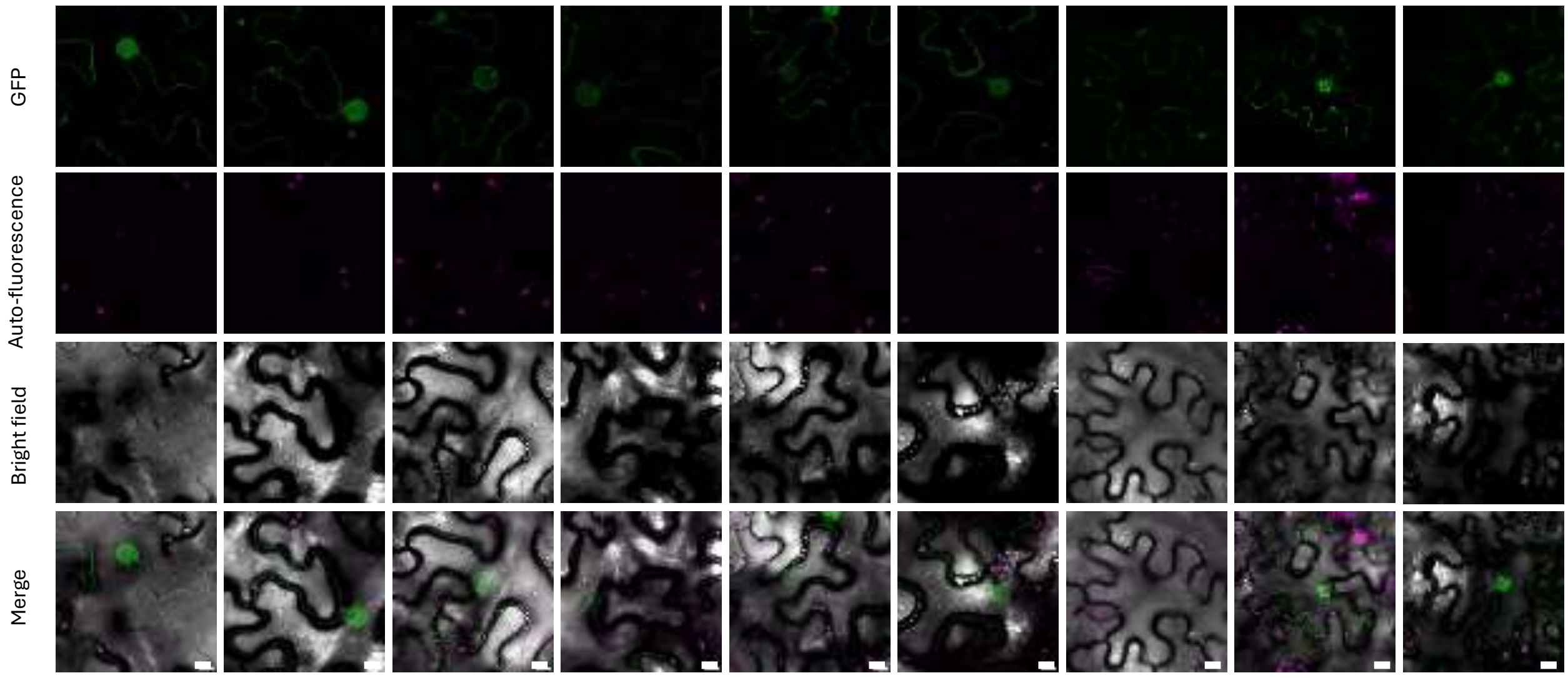

Supplementary figure 6. Subcellular localization of OELCuV C4-GFP in *N. benthamiana* leaves. *N. benthamiana* leaves infiltrated with *A. tumefaciens* carrying a construct to express OELCuV C4-GFP. Images were taken at 2 days post-inoculation (dpi). Scale bar: 10 μm.

SUPPLEMENTARY FIGURE 7

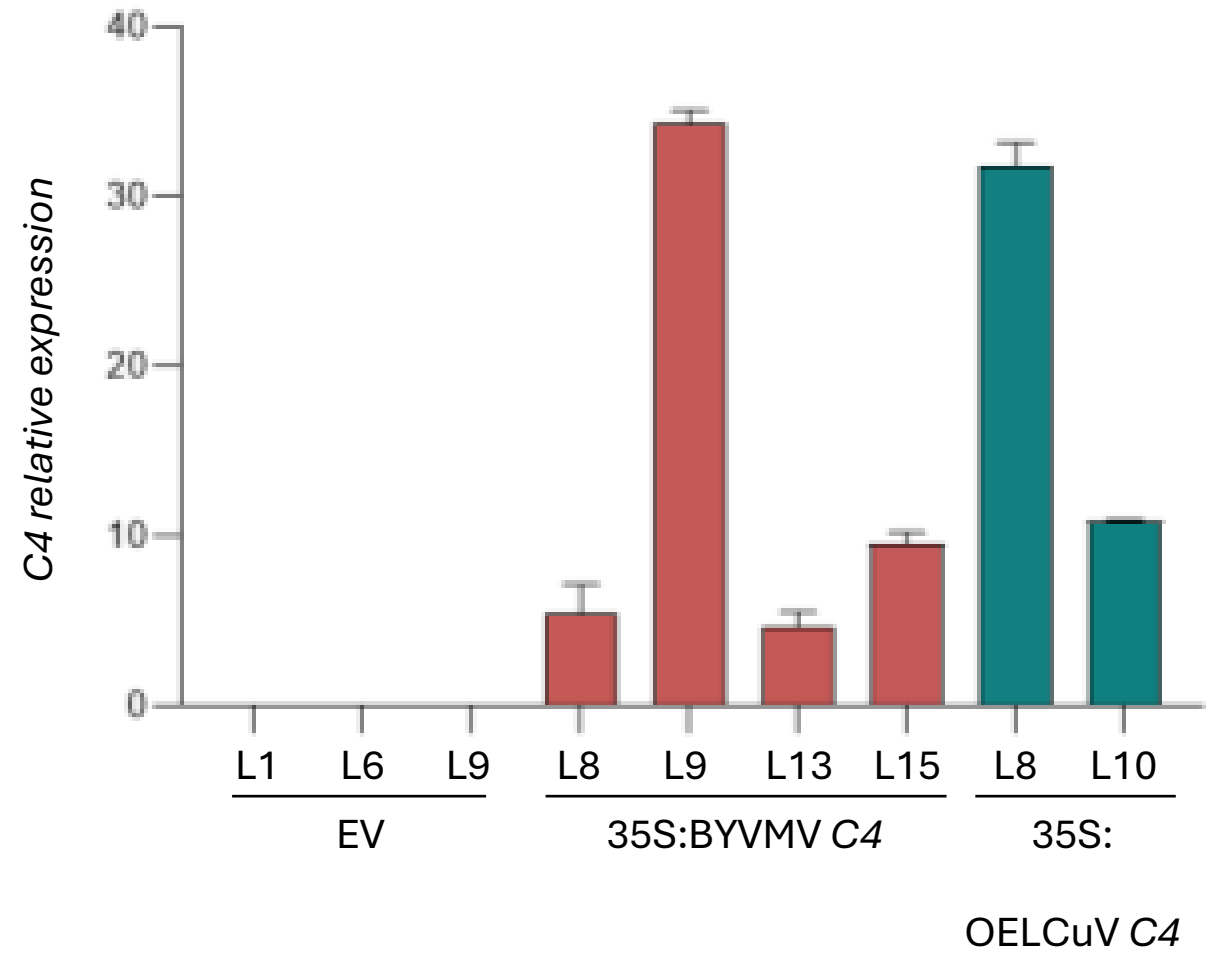

Supplementary figure 7. C4 expression in transgenic Arabidopsis plants. Accumulation of BYVMV or OELCuV C4 RNA in transgenic plants relative to actin mRNA, as measured by qRT-PCR. Each bar represents the mean of five 2-week-old seedlings from each independent line. Error bars represent SEM.

SUPPLEMENTARY FIGURE 8

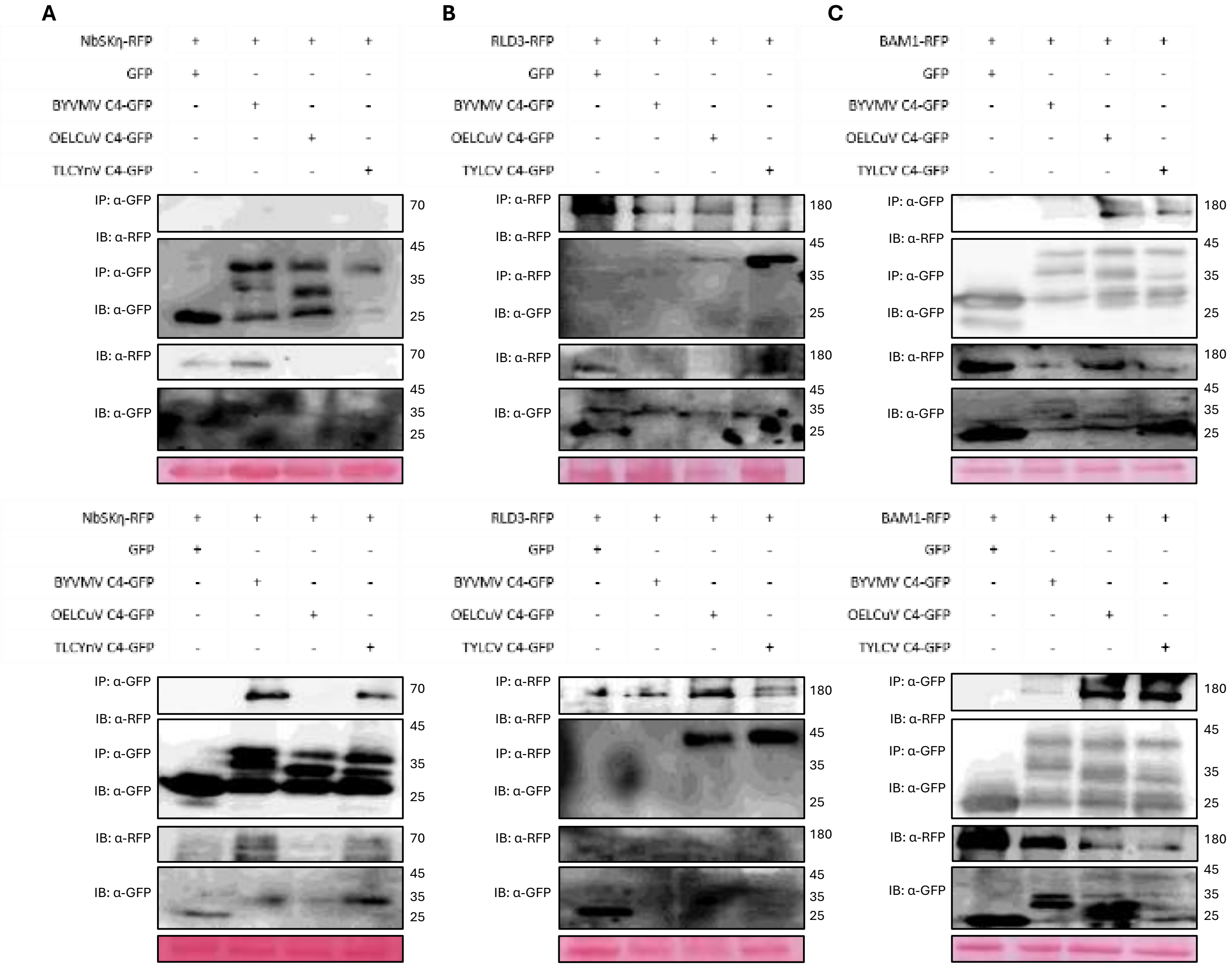

Supplementary figure 8. Additional replicates of co-immunoprecipitation (co-IP) assays from Figures 5 and 7. (a-c) Co-immunoprecipitation assays of GFP-fused BYVMV or OELCuV C4 with NbSKη- (a), RLD3- (b) and BAM1- RFP (c).
